# Effects of biofilm transfer and electron mediators transfer on *Klebsiella quasipneumoniae* sp. 203 electricity generation performance in MFCs

**DOI:** 10.1186/s13068-020-01800-1

**Published:** 2020-09-21

**Authors:** Yating Guo, Guozhen Wang, Hao Zhang, Hongyu Wen, Wen Li

**Affiliations:** School of Life Sciences, Jiangsu Normal University, 101 Shanghai Road, Xuzhou, 221116 Jiangsu China

**Keywords:** Microbial fuel cells, Electricity generation performance, Extracellular electron transfer, Biofilm, Electron mediators

## Abstract

**Background:**

Extracellular electron transfer (EET) is essential in improving the power generation performance of electrochemically active bacteria (EAB) in microbial fuel cells (MFCs). Currently, the EET mechanisms of dissimilatory metal-reducing (DMR) model bacteria *Shewanella oneidensis* and *Geobacter sulfurreducens* have been thoroughly studied. *Klebsiella* has also been proved to be an EAB capable of EET, but the EET mechanism has not been perfected. This study investigated the effects of biofilm transfer and electron mediators transfer on *Klebsiella quasipneumoniae* sp. 203 electricity generation performance in MFCs.

**Results:**

Herein, we covered the anode of MFC with a layer of microfiltration membrane to block the effect of the biofilm mechanism, and then explore the EET of the electron mediator mechanism of *K. quasipneumoniae* sp. 203 and electricity generation performance. In the absence of short-range electron transfer, we found that *K. quasipneumoniae* sp. 203 can still produce a certain power generation performance, and coated-MFC reached 40.26 mW/m^2^ at a current density of 770.9 mA/m^2,^ whereas the uncoated-MFC reached 90.69 mW/m^2^ at a current density of 1224.49 mA/m^2^. The difference in the electricity generation performance between coated-MFC and uncoated-MFC was probably due to the microfiltration membrane covered in anode, which inhibited the growth of EAB on the anode. Therefore, we speculated that *K. quasipneumoniae* sp. 203 can also perform EET through the biofilm mechanism. The protein content, the integrity of biofilm and the biofilm activity all proved that the difference in the electricity generation performance between coated-MFC and uncoated-MFC was due to the extremely little biomass of the anode biofilm. To further verify the effect of electron mediators on electricity generation performance of MFCs, 10 µM 2,6-DTBBQ, 2,6-DTBHQ and DHNA were added to coated-MFC and uncoated-MFC. Combining the time–voltage curve and CV curve, we found that 2,6-DTBBQ and 2,6-DTBHQ had high electrocatalytic activity toward the redox reaction of *K. quasipneumoniae* sp. 203-inoculated MFCs. It was also speculated that *K. quasipneumoniae* sp. 203 produced 2,6-DTBHQ and 2,6-DTBBQ.

**Conclusions:**

To the best of our knowledge, the three modes of EET did not exist separately. *K. quasipneumoniae* sp.203 will adopt the corresponding electron transfer mode or multiple ways to realize EET according to the living environment to improve electricity generation performance.

## Background

MFCs are kind of microbial electrochemical systems that use EAB in the anode as catalysts to convert chemical energy from organic matter into electricity [1–3]. Moreover, both electrodes of MFCs and Fe(III) oxides can act as extracellular electron receptors that receive electrons generated during EAB metabolism and are reduced. Most of the DMR bacteria in nature have strong electrochemical activity and can be used as anodic active bacteria in MFCs [4, 5]. Many previous studies proved that the operation of mixed communities have more significant advantages in electricity generation performance than the individual microorganism culture [6, 7]. However, the electricity generation process of mixed communities is more complicated. To understand the electricity generation mechanism of mixed communities, we must first clarify the electricity generation mechanism of the individual microorganism. In addition, clarification of the EET mechanism was considered to be an important factor to improve the power generation performance of EAB in MFCs [8]. Therefore, it was still worth paying attention to which EET of the individual microorganism can effectively improve power generation performance.

EET mechanism of EAB in MFCs can be summarized as follows: Intracellular electrons are transferred from the quinone pool on the inner membrane to the outside of the cell through the periplasm and outer membrane, during which it passes through a series of transmembrane cytochrome complexes. Electrons pass directly to the anode, which is the first EET mechanism [9]. The second EET mechanism is that after the electrons pass through the transmembrane cytochrome complex, the electrons are not directly transferred to the anode, but are transferred to the anode through conductive pilus, that is, nanowires [10, 11]. The above two mechanisms are due to the short distance electron transfer mechanism caused by the adsorption and growth of a large number of electrochemically active bacteria on the anode, which we can also call the biofilm mechanism. EAB can form a thick biofilm on the anode vertically or horizontally through the extracellular polymorphic substances (EPS). The biofilm is mainly composed of microbial cells adhering to the surface of the electrode and EPS. M. Islam et al. evaluated the effect of biofilm formation on power generation over time and found that *Klebsiella variicola* has a single layer and discretely distributed effective biofilm on the anode carbon brush of the double chamber MFCs to produce high power corresponding to low charge transfer resistance [12]. The third is the electron mediator mechanism: EAB, mainly DMR bacteria, can secrete low-molecular-soluble substances with redox activity and can be used as mediators for long-range electron transfer [8, 13]. The mechanism also makes EAB get rid of the limitation of direct contact with extra cellular electron receptors. Electron mediators are mainly involved in two redox processes in MFCs. (1) Bio-metabolism in biofilms, the electron mediator in an oxidized state obtains an electron to form the electron mediator in a reduced state; (2) The reduced electron mediator on the surface of the anode undergoes an electrochemical reaction, releasing the carried electrons and being anodized to an electron mediator in an oxidized state [14]. Electron mediators play an important role in bioenergy metabolism as intracellular electron carriers,for example, NAD mainly transfers electrons from the tricarboxylic acid cycle to the electron-transport chain, and membrane-bound terpenoids are primarily transmitted in respiratory protein complexes [15]. Immobilization of redox mediator on the surface of the anode is an effective way to improve the efficiency of extracellular electron transport and increase power output [16].

Currently, the EET mechanisms of DMR model bacteria *Shewanella oneidensis* and *Geobacter sulfurreducens* have been thoroughly studied [17]. However, the EET mechanism of *Klebsiella* has not been perfected. Through understanding the EET mechanism of *K. quasipneumoniae* sp. 203 in MFCs, it provides a theoretical basis for improving its electricity generation performance. Herein, we covered the anode of MFCs with a layer of microfiltration membrane to block the effect of the biofilm mechanism, and then explore the EET of the electron mediator mechanism of *K. quasipneumoniae* sp. 203 and electricity production performance. This study suggested that the three modes of EET did not exist in isolation. *K. quasipneumoniae* sp. 203 will adopt the corresponding electron transfer mode or multiple ways to realize EET according to the living environment.

## Results and discussion

### Preliminary verification of the electricity generation performance in coated-MFC and uncoated-MFC

**Fig. 1:**
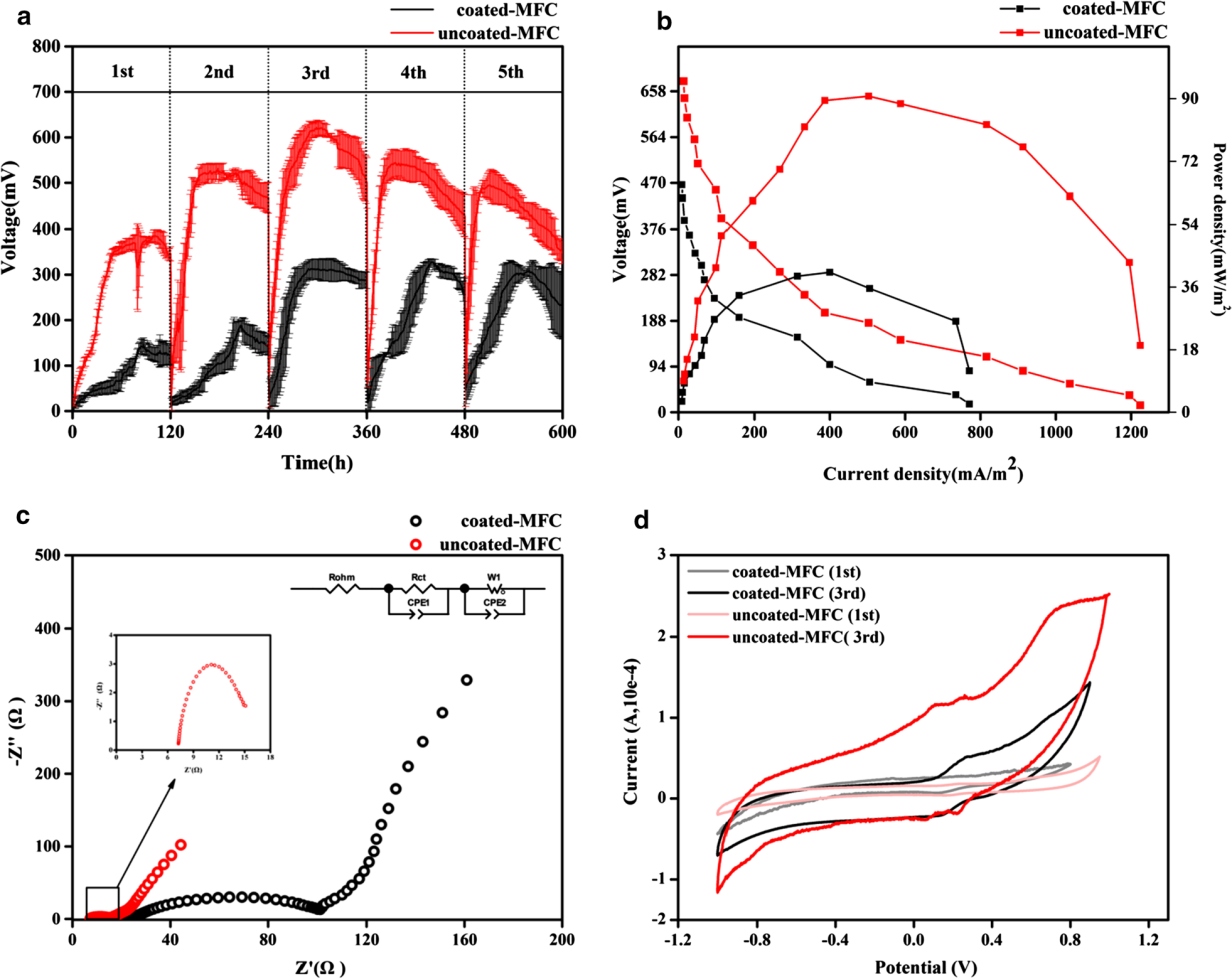
**a** MFC-coated and MFC-uncoated were incubated for 600 h of output voltage, and the anode medium was replaced when the output voltage droped. **b** Polarization curve and power density curve when the output voltage of the reaches its maximum value. **c** Nyquist plots from electrochemical impedance spectroscopy measurements of MFC-coated (black empty circle) and MFC-uncoated (red empty circle) scanned at 0.1 ~ 100 kHz at open-circuit potential (the inset on the right is the charge transfer resistance in the high frequency region of MFC-uncoated). **d** Cyclic voltammograms curve of coated-MFC and uncoated-MFC

Figure 1a shows that 5 cycles of *K. quasipneumoniae* sp. 203-inoculated MFCs were observed, a total of 5 cycles, each cycle lasted approx 120 h. Due to the consumption of the substrate sodium citrate in the anolyte, the output voltage will start to decline, and it is necessary to replace the anolyte. The average maximum output voltage in coated-MFC and uncoated-MFC were detected with 621 mV and 327 mV, respectively. Although the average maximum output voltage of coated-MFC was 300 mV lower than that of uncoated-MFC, it can be seen from Fig. 1b that coated-MFC still showed a certain electrochemical performance, coated-MFC reached 40.26 mW/m^2^ at a current density of 770.9 mA/m^2^ and the uncoated-MFC reached 90.69 mW/m^2^ at a current density of 1224.49 mA/m^2^. The difference in the electricity generation performance between coated-MFC and uncoated-MFC was probably due to the microfiltration membrane covered in anode, which inhibited the growth of EAB on the anode. Therefore, we speculated that *K. quasipneumoniae* sp. 203 can also perform EET through the biofilm mechanism. C. Yuvraj et al. also indicated that *K. quasipneumoniae* can directly transfer electrons to the anode without any external mediator, and the increase in electrochemical performance is directly proportional to the electroactive biofilm formed on the electrode surface [18]. *Shewanella* and *Geobacter* are considered model exogenous electrons, and are known to be able to perform direct extracellular electron transitions through outer membrane redox proteins [6]. According to previous studies, the common feature of EAB with this EET ability is the presence of many polyheme c-type cytochromes (MH-cytC) in their genome [19].

Figure 1c presents that the internal resistance of the MFCs, the semicircles in the high frequency region and the straight lines in the low frequency region represent the charge transfer resistance (Rct) and diffusion resistance (W_1_) of the MFCs, respectively. The *R*_ct_ and W_1_ of coated-MFC were 78.20 Ω and 55.10 Ω, the *R*_ct_ and W_1_ of uncoated-MFC were 6.84 Ω and 30.20 Ω, the *R*_ct_ and *W*_1_ of coated-MFC were both higher than uncoated-MFC. After the operation of MFCs, the anode biofilm continued to grew and generate electrons until a complete biofilm was formed on the anode surface, and the electricity generation performance of the system reached a stable state. For EAB, the phospholipid bilayer structure of the cell membrane acts as a capacitor, and the electron shuttle (or redox mediator) generated endogenously on the cell membrane acts as an electrochemical active site. The surface of the coated-MFC anode was covered with a microfiltration membrane. It was difficult for microbial cells to adhere to the smooth surface of the microfiltration membrane, and the electrons generated by it cannot be transmitted to the anode surface over a long distance without electron mediators [7]. It is generally believed that, within a certain period of time, the thickness of the anode biofilm and the content of the redox mediator are negatively related to the internal resistance [20]. MFCs performance metrics were summarized in Table 1 and the data indicated that the output power of MFCs is inversely proportional to the internal resistance.

**Table 1:** Comparison of electricity generation performance and internal resistance in coated-MFC and uncoated-MFC

	Power density (mW/m^2^)	Current density (mA/m^2^)	Internal resistance (Ω)				
			Rohm	Rct	W_1_	CPE1	CPE2
Coated-MFC	40.26	770.97	23.54	78.20	55.10	1.76	2.74
Uncoated-MFC	90.69	1224.49	7.41	6.84	30.20	7.20	8.61

Figure 1d shows the electron transfer mechanism and catalytic efficiency during the stable stage of 1st and 3rd operation of the MFCs. The CV curve revealed that no significant redox peaks were observed in 1st cycle of coated-MFC and uncoated-MFC. After 3rd cycle of operation, the existence of a reversible redox process in both MFCs, but the peak of coated-MFC was significantly lower than that of uncoated-MFC, and more than one pair of redox peaks of uncoated-MFC can be observed. In 3rd cycle of coated-MFC, a redox peak that was not detected in the 1st operation was observed. Therefore, it can be inferred that the electrochemical activity of coated-MFC due to the self-excreted electron mediators lead to the mechanism of electron transfer from bacterium to the anode. A similar result was also obtained from the analysis by Deng et al. [8]. Interestingly, uncoated-MFC showed more intense redox activity than coated-MFC, indicating that uncoated-MFC can also transfer electrons through other EET mechanisms in addition to generating electron mediators. Islam et al. also observed a similar redox peak from the MFCs inoculated with *Klebsiella variicola* [7]. Consequently, the appearance of more intense redox peaks indicated that the mature and effective biofilm was formed on the anode surface, which shortened the diffusion distance of extracellular electron transfer between EAB and the anode.

### Effect of microfiltration membrane on the growth of cells

**Fig. 2:**
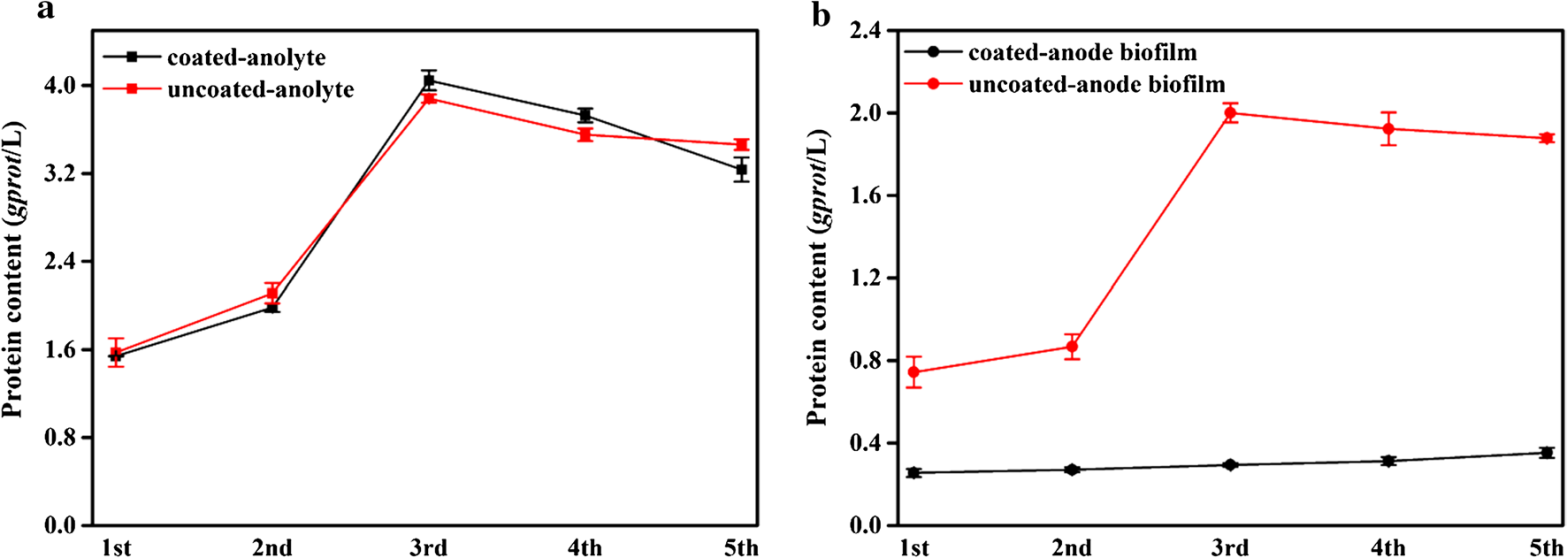
Protein contents of coated-MFC and uncoated-MFC. **a** Anolyte suspension; **b** Anode biofilm

We were interested in whether the microfiltration membrane affects cell growth, so we measured the biomass of the anolyte and anode. The biomass of the microbial population can be indirectly calculated by measuring the protein content [21]. During the electricity generation process of MFCs, the amounts of EAB in anode biofilm and anolyte suspension in coated-MFC and uncoated-MFC as assessed by the protein contents were compared (Fig. 2). We found that the protein content is basically the same in coated-MFC and uncoated-MFC, and the presence of microfiltration membrane has little effect on the anolyte suspension biomass (Fig. 2a). Moreover, we found that the biomass of uncoated-MFC anode biofilm was much higher than that of the coated-MFC, and the average protein content by more than 5 times (Fig. 2b). The existence of the microfiltration membrane only affected the biomass of the anode biofilm, and had little effect on the biomass in the anolyte. Therefore, it can be considered that the difference in the electricity generation performance between coated-MFC and uncoated-MFC was due to the extremely little biomass of the anode biofilm.

### Effect of anode biofilm on electricity generation performance of MFCs

**Fig. 3:**
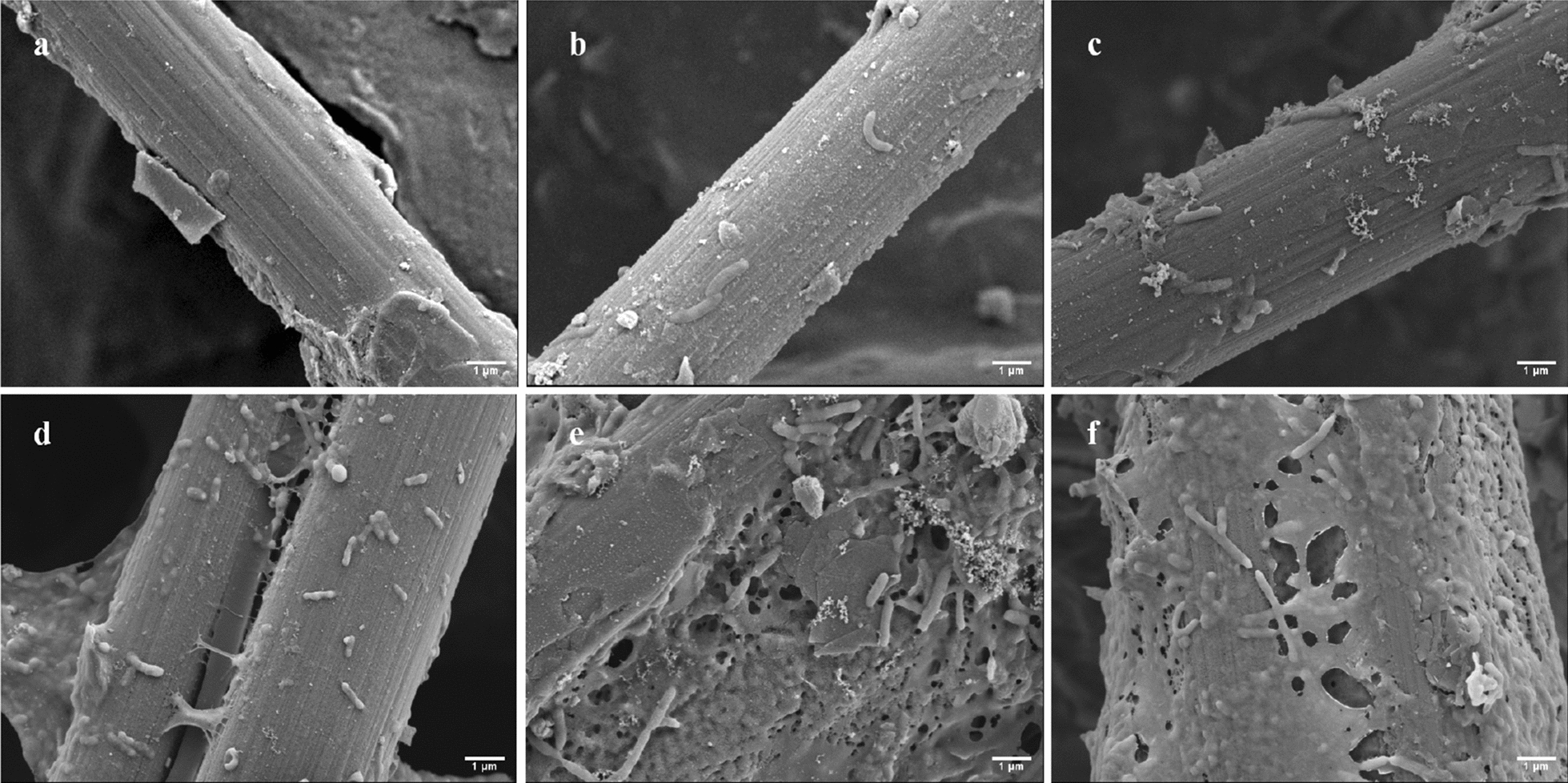
SEM images of anode biofilm of coated-MFC and uncoated-MFC, **a** coated-MFC: 1st cycle, **b** coated-MFC: 3rd cycle, **c** coated-MFC: 5th cycle **d** uncoated-MFC: 1st cycle, **e** uncoated-MFC: 3rd cycle, **f** uncoated-MFC: 5th cycle

Figure 3 reveals that the formation of the biofilm on anode electrode surface by SEM. Results clear showed that almost no EAB were attached to the carbon paper surface in coated-MFC (Fig. 3a–c). This further verified the existing results, the extremely low protein content of coated-MFC anode biofilm (Fig. 2b). However, the adsorption capacity of rod-shaped EAB at the anode increased with time in uncoated-MFC (Fig. 3d–f). It can be seen that an incomplete biofilm is formed on the electrode surface due to the adsorption of EAB in the 1st operation of uncoated-MFC (Fig. 3d). It was not until the third operation of uncoated-MFC that we observed that the microorganisms attached to the surface of the carbon paper began to secrete an enveloping matrix consisting mainly of polysaccharides and proteins, known as EPS. According to the research by Wu et al., EPS can accumulate group-sensing effect signaling molecules, extracellular enzymes, and bacterial secondary metabolites, providing a place for microbial to exchange information [22]. Kim et al. tested the impedance of the biofilm formation process in the early adhesion stage of *P. aeruginosa* PAO1 and found that early adhesion of microorganisms on the anode would lead to a reduction in electrical resistance [23]. In combination with the above electrochemical performance results, we considered that microorganisms clusters form complete biofilms with metabolic activity, thereby exhibiting higher power generation performance (Figs. 1a, 3e). However, in the 5th cycle, several layers of biofilm were adsorbed on the anode of uncoated-MFC which decreased the electrochemical performance (Fig. 3f). This result was consistent with the result of protein content of coated-MFC anode biofilm (Fig. 2b). In the 3rd cycle of uncoated-MFC, the output voltage (621 mV) and the protein content anode biofilm (2.00 g*prot*/L) reached their peak values at the same time (Fig. 1a). And then the protein content decreased with the output voltage - time. This may be related to the EAB proliferation rate of anode biofilm.

**Fig. 4:**
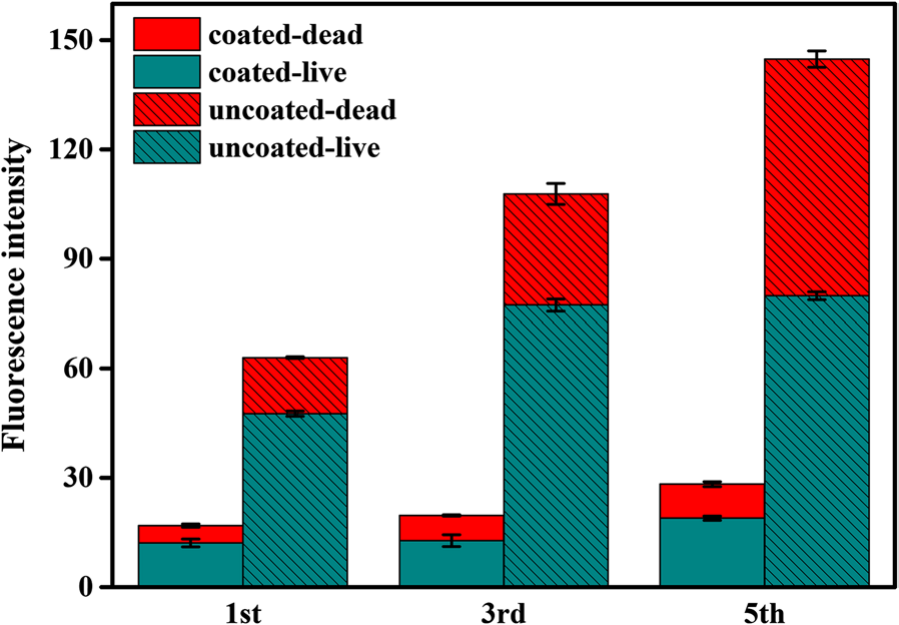
Fluorescence intensity of live and dead cells of coated-MFC and uncoated-MFC

In addition, the changes in biofilm viability were examined over time using fluorescent staining to distinguish live versus dead cells (Fig. 4 and Additional file 1: Fig. S1). As previously described, the presence of microfiltration membrane maked it almost impossible to observe the presence of living and dead cells (Additional file 1: Fig. S1a–c). The fluorescence intensity of dead cells was much less than that of living cells (Fig. 4). On the contrary, as the biofilm grew, cells existed in two states (live and dead) in uncoated-MFC (Additional file 1: Fig. S1d–f). When the maximum output voltage reached 621 mV in the 3rd cycle, we observed that the entire biofilm of *K. quasipneumoniae* sp. 203 was alive, with very few dead cells (Fig. 1a and Fig. S1e). As the biofilm grew, the fluorescence intensity of red dead cells increased from 30.49 to 64.95, the increased fluorescence intensity of dead cells can help explain that the output voltage gradually decreases after reaching the peak (Fig. 4, Additional file 1: Fig. S1e, f). Although we did not analyze the location and spatial structure of live and dead cells in biofilm, previous studies suggested that living cells can only exist in the outer layer of thick biofilm, which may be due to the availability of anode electrolyte substrates [24, 25]. Furthermore, the accumulation of dead cells (more than active cells) at the bottom of the biofilm over time will not exert redox activity. Thus this increased the intrinsic resistance of MFCs.

### Effect of electron mediators on electricity generation performance of MFCs

The model strains for EET mechanism were *Geobacter sulfurreducens* and *Shewanella oneidensis*. *G. sulfurreducens* can also synthesize a small amount of flavin compound, but it can only binds to the outer membrane protein and cannot be released as an electron mediator, like *S. oneidensis*. Therefore, it is generally believed that *G. sulfurreducens* undergoes short-range electron transfer by direct contact with extracellular electron acceptors [5]. Combined with this study, it was found that when a new anode medium was replaced at the end of each cycle, the output voltage decreased significantly and rose slowly, even over 24 h, which was in sharp contrast to *G. sulfurreducens* [26].

**Fig. 5:**
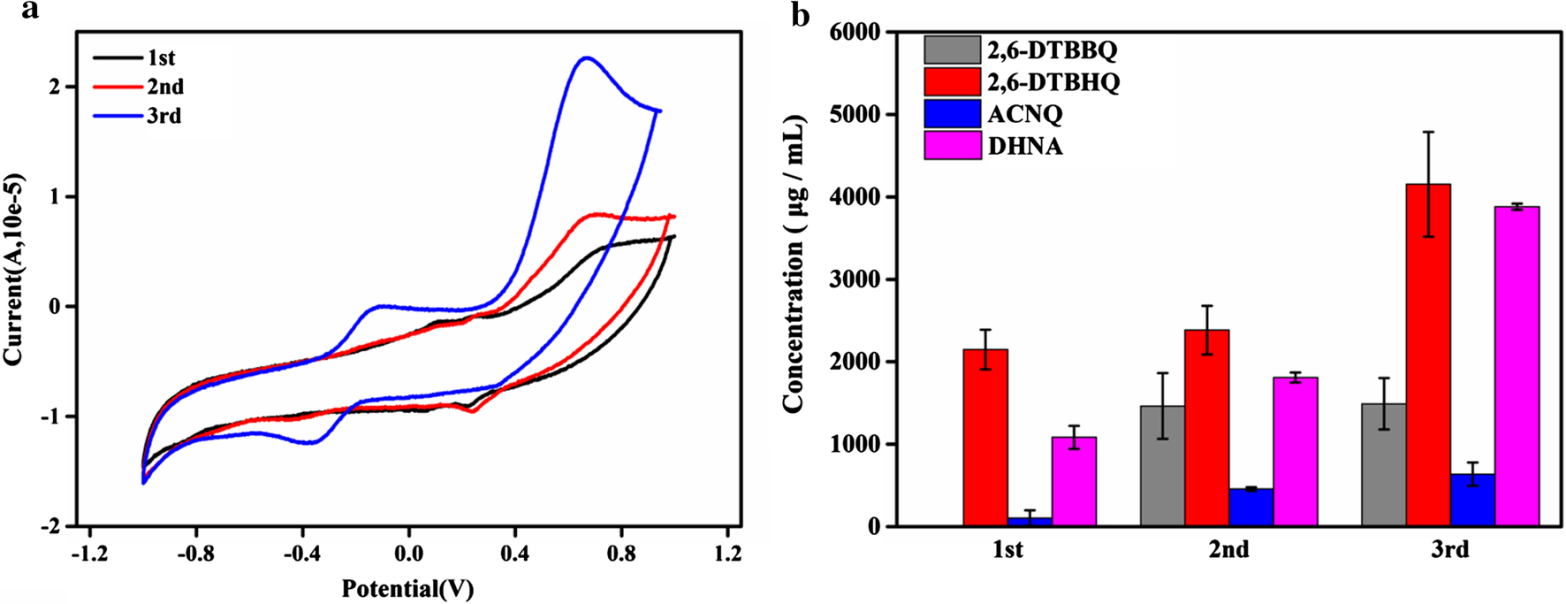
**a** CV of the supernatant of the anode on the 1–3 cycle, **b** Quinones electron mediators secreted by *K. quasipneumoniae* sp. 20

Therefore, compared with the CV curve of coated-MFC, we found that a pair of redox peak that appeared in the range of − 0.4 ~ 0 V in the CV curve of the supernatant did not appear in the MFCs (Figs. 1d, 5a). According to the report by Freguia et al., it may be due to EAB actively removing mediators in an oxidized state [27]. To better detect the electron mediators, we conduct CV detection on the anode supernatant. As shown in Fig. 5a, we found that more than one pair of redox peaks appeared in the anode supernatant. By comparing the standard library and consulting related references, it is inferred that the possible quinone electron mediators secreted by *K. quasipneumoniae* sp.203 were 2,6-di-tert-butyl-p-benzoquinone (2,6-DTBBQ), 2,6-Di-tert-butylphenol (2,6-DTBHQ), 1,4-dihydroxy-2-naphthoic acid (DHNA) and 2-amino-3carboxy-1,4-naphthoquinone(ACNQ) (Fig. 5b, Additional file 1: Tables S1 and S2) [14, 28, 29].

**Fig. 6:**
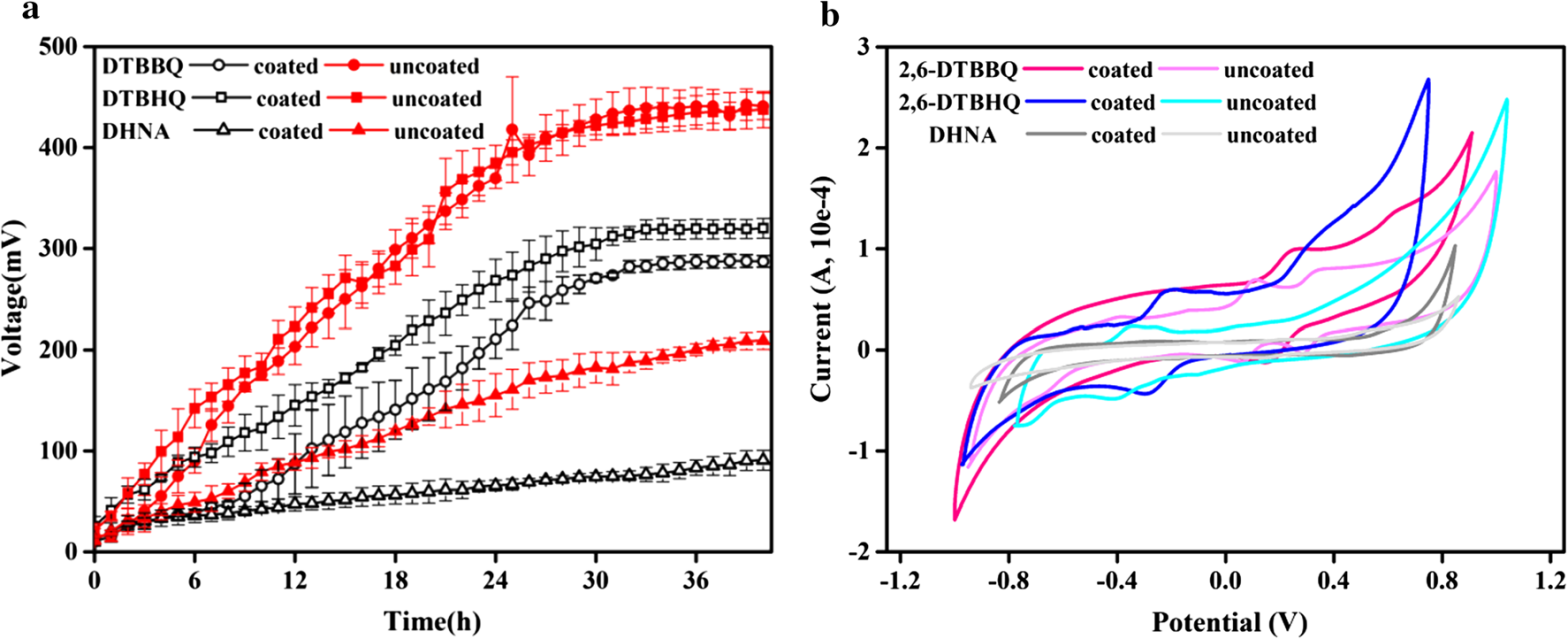
Effect of three quinone electron mediators secreted by *K. quasipneumoniae* sp. 203 on electricity generation performance of MFCs. **a** The output voltage–time; **b** Cyclic voltammogram curve

Herein, combined with the existing research ideas of this experiment, MFCs constructed by *K. quasipneumoniae* sp. 203 was used as the research object, upon the addition of 10 µM 2,6-DTBBQ, 2,6-DTBHQ and DHNA to the reactor, respectively, and the results showed that the voltage of coated-MFC and uncoated-MFC increased immediately, except for the addition DHNA of MFCs (Fig. 6a). Although the voltage of the coated-MFC was lower than that of the uncoated-MFC, the voltage reached the highest output voltage within 40 h. The sharp rise in the voltage proved that the 2,6-DTBBQ and 2,6-DTBHQ can function as electron mediators in MFCs, thus facilitating the electron transfer from EAB to electrode. This speculation was similar to the results of Deng et al. [30]. HPLC-MS detected a low content of ACNQ, so we did not add ACNQ to the reactors.

We also performed CV to examine the redox state of MFCs after the addition of electron mediators (Fig. 6b). The redox peaks appeared in coated-MFC and uncoated-MFC after adding 2,6-DTBBQ and 2,6-DTBHQ. Notably, the pair of redox peak at 0 ~ 0.8 V were attributed to 2,6-DTBBQ, and the pair of redox peak at − 0.4 ~ 0 V were assigned to 2,6-DTBHQ. This result almost corresponded to the CV curve of the supernatant (Fig. 5a). In contrast, no redox peaks were found in the addition DHNA of MFCs. Combined with the time–voltage curve, we also found that after adding DHNA, the voltage of the MFC did not show a significant upward trend. Therefore, we speculate that DHNA was not a redox metabolite of *K. quasipneumoniae* sp. 203.

Combining the time–voltage curve and CV curve, we found that 2,6-DTBBQ and 2,6-DTBHQ had high electrocatalytic activity toward the redox reaction of *K. quasipneumoniae* sp. 203-inoculated MFCs. Moreover, the oxidation and reduction peaks at the range of − 0.4 ~ 0 V correspond to 2,6-DTBBQ, 2,6-DTBHQ, this result was similar to that of Zeng. et al. in detecting the electrocatalytic activity of *Klebsiella pneumoniae* on 2,6-DTBBQ [31]. This contributes to the electron transfer between the EAB and the MFCs anode and also contributes to the power density of the MFCs.

According to previous studies, the electron mediators-mediated extracellular electron-transport mechanism is a circulation mechanism. The oxidized mediators are converted into the reduced mediators after being coupled intracellularly with the reduction products on the respiratory chain [9]. Then reduced mediators are discharged out of the extracellular, and the electrons are transferred to the electrode to be oxidized. It is speculated that the electron mediator secreted by *K. quasipneumoniae* sp. 203 can be reused. In the stable growth stage of anode EAB in MFCs, the addition of electron mediators can effectively improve the electricity generation performance. Quinones can be used as electron mediators, mainly because of their quinone group with electron transfer function. Based on the existing research of this experiment, it is considered that the transfer mechanism of the electron mediators in coated-MFC and uncoated-MFC is: the quinone compounds secreted by EAB were obtained electrons and reduced into hydroquinones, and then electrons were transferred to the anode, and hydroquinones were oxidized to benzoquinones. The main role of quinones as electron mediators is due to their quinone group with electron transfer function. The group is circulated intracellularly in three states: oxidized, semiquinone radical, reduced or hydroquinone. The transition of the electron mediator from the oxidized state to the reduced state is accomplished by quinone reductases under the action of flavin adenine dinucleotide (FAD) [28]. And the studies by Ramos et al. has shown that the redox mediators secreted by EAB can also promote the formation of biofilms [32].

## Conclusions

In this study, the coated-MFC anode was covered with a microfiltration membrane to investigate whether *K. quasipneumoniae* sp. 203 can conduct EET through the electron mediator mechanism. In the absence of short-range electron transfer, we found that *K. quasipneumoniae* sp. 203 can still produce certain electricity generation efficiency and redox activity. The protein content, the integrity of biofilm and the biofilm activity all proved that the existence of microfiltration membrane prevented EAB from attaching and growing on the anode, resulting in the difference in the electricity generation performance between coated-MFC and uncoated-MFC. Finally, to further prove the effect of electron mediators on the performance of electricity generation, combining the time–voltage curve and CV curve of MFCs after adding electron mediators, we found that 2,6-DTBBQ and 2,6-DTBHQ had high electrocatalytic activity toward the redox reaction of *K. quasipneumoniae* sp. 203-inoculated MFCs. Our work demonstrated that *K. quasipneumoniae* sp. 203 can be coupled to realize EET in a variety of ways in MFCs. Through understanding the EET mechanism of *K. quasipneumoniae* sp. 203 in MFCs, it provides a theoretical basis for improving its power generation performance.

## Methods

### Strain and culture condition

A EAB for this experiment was obtained from the anode biofilm sample of a constructed wetland-microbial fuel cell (CW-MFC), and the separating and purifying method of the bacteria was as described by Wang et al. [33]. The strain was identified as *K. quasipneumoniae* sp. 203 by 16rRNA gene sequencing,and the accession number in Genbank is MN900629. The strain was stored in China General Microbiological Culture Collection Center (CGMCC) under the accession No. 19001. *K. quasipneumoniae* sp. 203 was grown in 10 mL Luria–Bertani (LB) liquid medium previously. Hereafter, the defined media (DM) medium was used for preculture which contained the following components(per liter of distilled water): 0.1 g KCl, 0.25 g NH_4_Cl, 0.05 g KH_2_PO_4_, 0.015 g MgCl_2_, 0.015 g CaCl_2_, 3 µL of trace elements solution and 3 µL of vitamin solution. The two medium both in a shaking incubator at 31 °C with 200 rpm until the OD_600_ of the pre-cultured bacterium reached 1.0 (Spectrophotometer UV 7504, Shanghai, China). After incubation, bacteria precipitation was obtained by centrifugation at 5000 rmp for 10 min.

### MFCs construction and operation

**Fig. 7:**
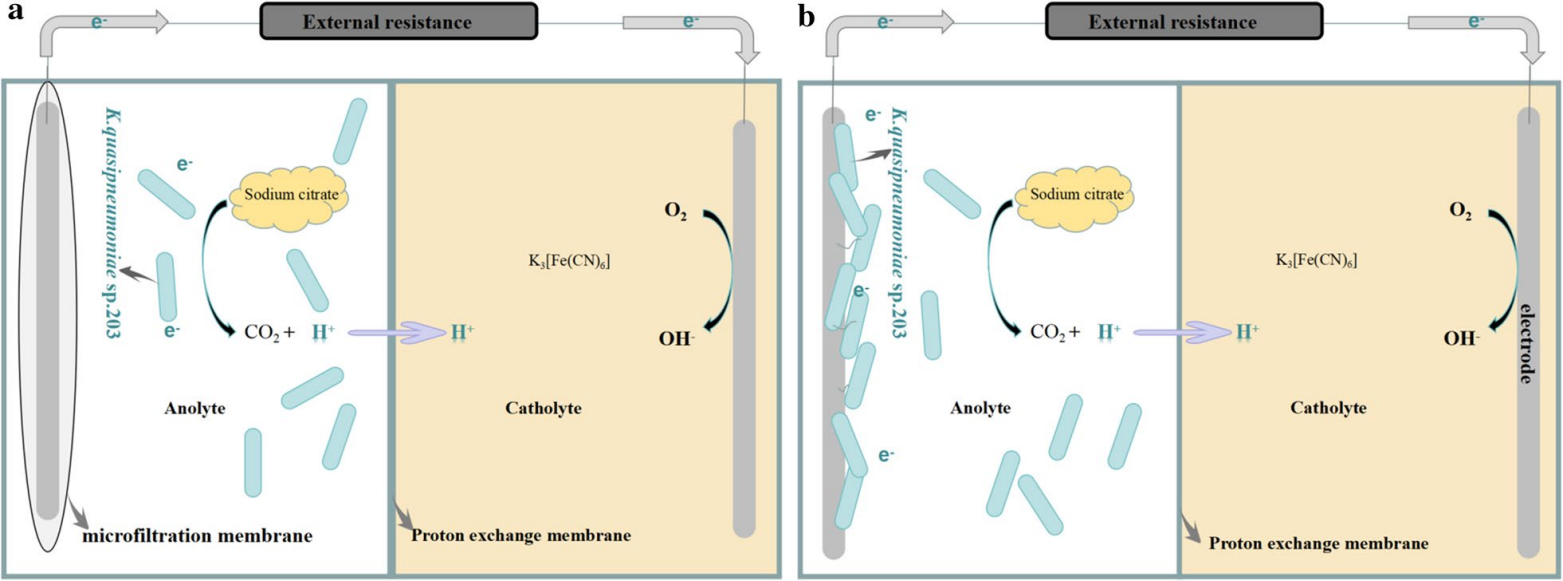
Schematic diagram of the anode chamber of MFCs. **a** coated-MFC, **b** uncoated-MFC

Two double-chamber MFCs reactors were constructed by cubic plexi glasses (an anode chamber and a cathode chamber), and the total working volume of each chamber of 50 mL. Porous carbon paper with a diameter of 2.5 cm used as the anode and the cathode electrode. The anode electrode of coated-MFC was coated with a microfiltration membrane with a pore size of 0.22 μm (avoid microbial cells passing) and uncoated-MFC was untreated. The schematic diagram of the anode chamber of MFCs is shown in Fig. 7. The proton exchange membrance (Nafion 117, USA, 183 µm) separates the cathode chamber from the anode chamber, prior to experiment, the cation exchange membrane was treated with 1 M HCl and then rinse with distilled water. The anolyte was DM medium with sodium citrate as the sole carbon source at the final concentration of 20 mM. K_3_Fe (CN) _6_ (50 mM) solution in PBS (0.1 M, PH = 7.4) was used in the cathode chamber. Before experimental, the components of MFCs sterilized by autoclaving at 121 °C for 20 min. MFCs connected a 2000 Ω external resistor.

### Electrochemical measurements

The time voltage date of MFCs was monitored by signal acquisition card (8AD PLUS, Yav, Wuhan, China). The electrochemical data was detected by electrochemical workstation (CHI660E, CH instruments, Shanghai, China). Cyclic voltammetry (CV) measurements were to detect electrochemical activity with a three electrode configuration. The anode and the cathode electrode were performed as the working electrodes and the counter electrode, respectively. The Ag/AgCl (KCl, 1.0 M) was sterilized with ethanol (70%) which used as the reference electrode and located near the anode electrode. CV measurements with a scan rate was 5 mV/s, the scanning potential range from + 1.0 V to − 1.0 V. The detection of Electrochemical impedance spectroscopy (EIS) is the same as CV, in the frequency range of 0.1–100 kHz. Using ZView software, the equivalent circuit was fitted to the impedance data, and the composition and size of the internal resistance of the MFCs were analyzed. Rohm, Rct, CPE, and W1 in the equivalent circuit represent ohmic resistance, charge transfer resistance, constant phase element, and diffusion resistance, respectively. Adjustment of the external resistance over a range of 50–100 kΩ to obtain the current density and the maximum power density (calculate by *P*_*s*_ (mW/m^2^) = *U*^2^/(*SR*), *I*_*s*_ = *U*/*SR*, *U*, output voltage; *S*, anode carbon paper area; *R*, external resistance).

### Biomass detection of anolyte suspension and anode biofilm

Bicinchoninic acid (BCA) Protein Assay Kit (Thermo Scientific) was used to determine the total protein content of the anolyte suspension and the anode biofilm of MFCs, so as to characterize the effect of microfiltration membrane modification on the total amount of microorganisms of the anolyte and attached to anode during the power generation process.

### Microscopic study of the anode biofilm

After a period of generating electricity, aggregation of microbial cells on the anode of the MFCs operation. To observe the thickness of the anode biofilm at different cycles, scanning electron microscope (SEM, SUPRA™55, Carl Zeiss AG, Germany) at 5 kV was used. The undetected sample was prepared essentially according to the method: a small portion of the anode covered with the biofilm is taken out from the anode chamber, and then cut into a block by a size of 1–2 cm. Thereafter treated with the following solutions: (1) The sample was immersed in sterile DM medium at 31 °C for 4 h. (2) 3% glutaraldehyde solution with the anaerobic conditions at 4 °C for 1 h. (3) 0.1 M phosphate buffer by 2 or 3 times. (4) Dehydration with different concentrations of ethanol (40%, 60%, 80%, 90% and 100%) by 10 min. Finally, the sample was dried and coated with platinum by ion sputtering to a thickness of up to 10 nm and the specimens were examined by SEM.

### Detection of cell viability of anode biofilm

The LIVE/DEAD^®^ BacLight™ Bacterial Viability Kit (Thermo Fisher Scientific) can be used to assess cell viability [7]. Bacteria are labeled according to the difference in permeability between dead and live bacteria. Under the excitation of argon laser, SYTO9 is bright green fluorescence, which characterizes live bacteria, PI is red fluorescence, which characterizes dead bacteria [34]. The cell viability of *K. quasipneumoniae* sp. 203 in different growth phases were visualized using Fluorescence Microscope and analyzed by the software of ImageJ-win64. Varioskan LUX (Thermo Fisher, Scientific) was used to measure the fluorescence intensity.

### Detection and analysis of electron mediators

To determine the electron mediators secreted by *K. quasipneumoniae* sp. 203 in MFCs, high performance liquid chromatography mass spectrometry (HPLC–MS, AB SCIEX 4600 Triple TOF, Co, Ltd, USA) was used for analysis. The anolyte samples were pretreated and the method was modified as described by Islam et al. [35]. The HPLC program parameters were similar to those described by Wang et al. [36]. The column temperature was kept at 35 °C. MS data was collected via an AB SCIEX TripleTOF 4600 mass spectrometer. The mass spectrometry parameters were: in ESI + mode, the ionization energy was set to 10 V, the cluster potential was set at 70 V, the electron multiplier voltage was 4500 V, and the mass-to-charge ratio (m/z) was selectively monitored from 50 to 1200.

## Supplementary information

**Additional file 1. Effects of bioilm transfer and electron mediators transfer on Klebsiella quasipneumoniae sp. 203 electricity generation performance in MFCs.**

## Data Availability

We declared that materials described in the manuscript, including all relevant raw data, will be freely available to any scientist wishing to use them for non-commercial purposes, without breaching participant confidentiality.

## Abbreviations

EET: extracellular electron transfer
EAB: electrochemically active bacteria
MFCs: microbial fuel cells
DMR: dissimilatory metal-reducing
EPS: extracellular polymorphic substances
CW-MFC: constructed wetland-microbial fuel cell
CGMCC: China General Microbiological Culture Collection Center
LB: Luria–bertani
DM: defined media
CV: cyclic voltammetry
EIS: electrochemical impedance spectroscopy
Rct: the charge transfer resistance
Rd: the diffusion resistance
Rohm: the ohm resistance
SEM: scanning electron microscope
BCA: bicinchoninic acid
HPLC–MS: high performance liquid chromatography mass spectrometry
MH-cytC: many polyheme c-type cytochromes
2,6-DTBBQ: 2,6-di-tert-butyl-p-benzoquinone
2,6-DTBHQ: 2,6-Di-tert-butylphenol
DHNA: 1,4-dihydroxy-2-naphthoic acid
ACNQ: 2-amino-3carboxy-1, 4-naphthoquinone
FAD: flavin adenine dinucleotide
