# Supplementary material for: Effects of biofilm transfer and electron mediators transfer on Klebsiella quasipneumoniae sp. 203 electricity generation performance in MFCs
Source: Biotechnol Biofuels. 2020 Sep 21;13:162. doi: 10.1186/s13068-020-01800-1 (PMC7507662; doi:10.1186/s13068-020-01800-1)
Supplement: Supplementary file 1 — Additional file 1. Effects of bioilm transfer and electron mediators transfer on Klebsiella quasipneumoniae sp. 203 electricity generation performance in MFCs. [file 13068_2020_1800_MOESM1_ESM.doc]

| Cycle | 1st | | | | 2nd | | | | 3rd | | |
| --- | --- | --- | --- | --- | --- | --- | --- | --- | --- | --- | --- |
| 2,6-Di-tert- butylpbenzoquinone  (2,6-DTBBQ) | - | - | - | 1577 | | 1793 | 1720 | 1292 | | 1450 | 1330 |
| 2,6-Di-tert- butylphenol  (2,6-DTBHQ) | 1925 | 2044 | 1978 | 2044 | | 2555 | 2555 | 4444 | | 4834 | 4578 |
| 1,4-dihydroxy-2- naphthoic acid (DHNA) | 1222 | 942 | 1089 | 1752 | | 1872 | 1801 | 3912 | | 3842 | 3888 |
| 1,4-benerkun | + | - | - | - | | - | - | - | | - | - |
| 2-amino-3- dicarboxy-1,4- naphthoquinone (ACNQ) | 140 | 178 | 122 | 434 | | 475 | 464 | 598 | | 532 | 584 |

**Table S1** Eelectron mediators of quinones may be secreted by *K.quasipneumoniae* sp.203

**Table S2** The data of HPLC-MS

1st cycle


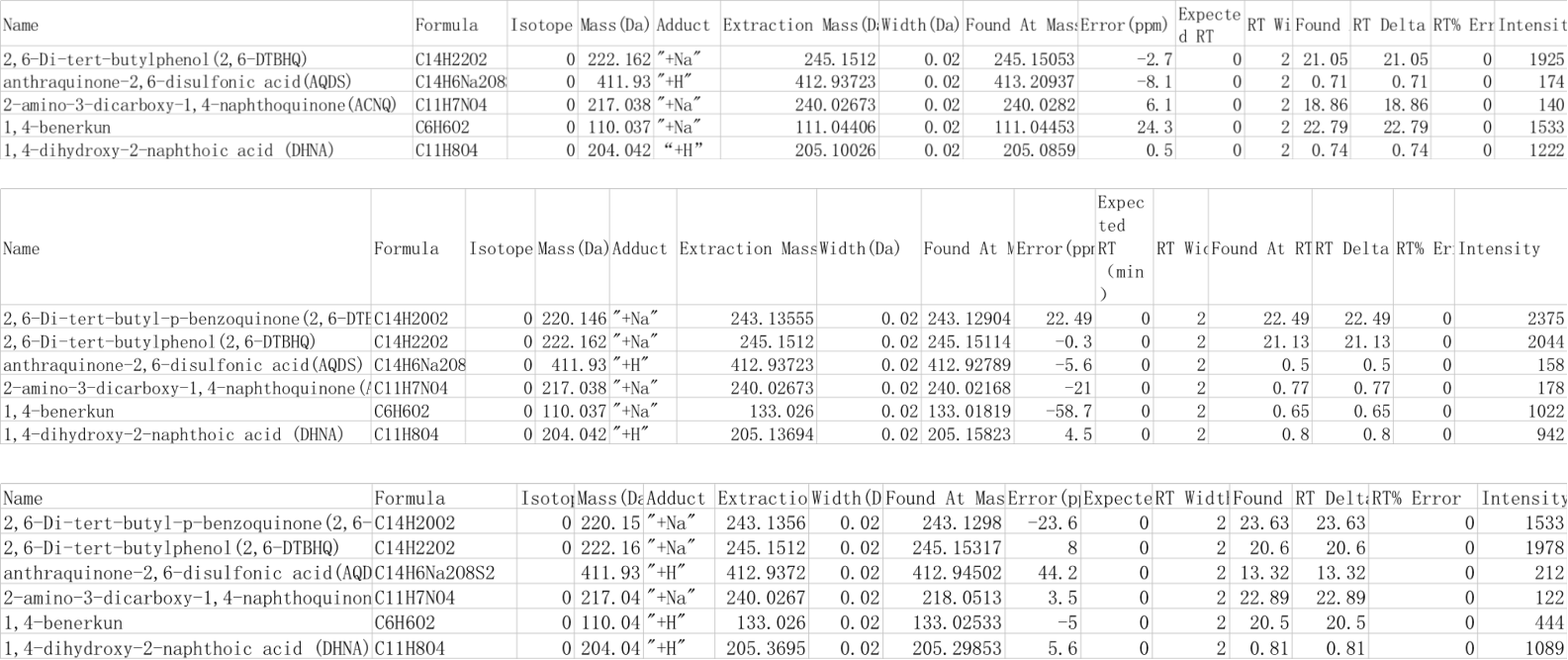


2nd cycle


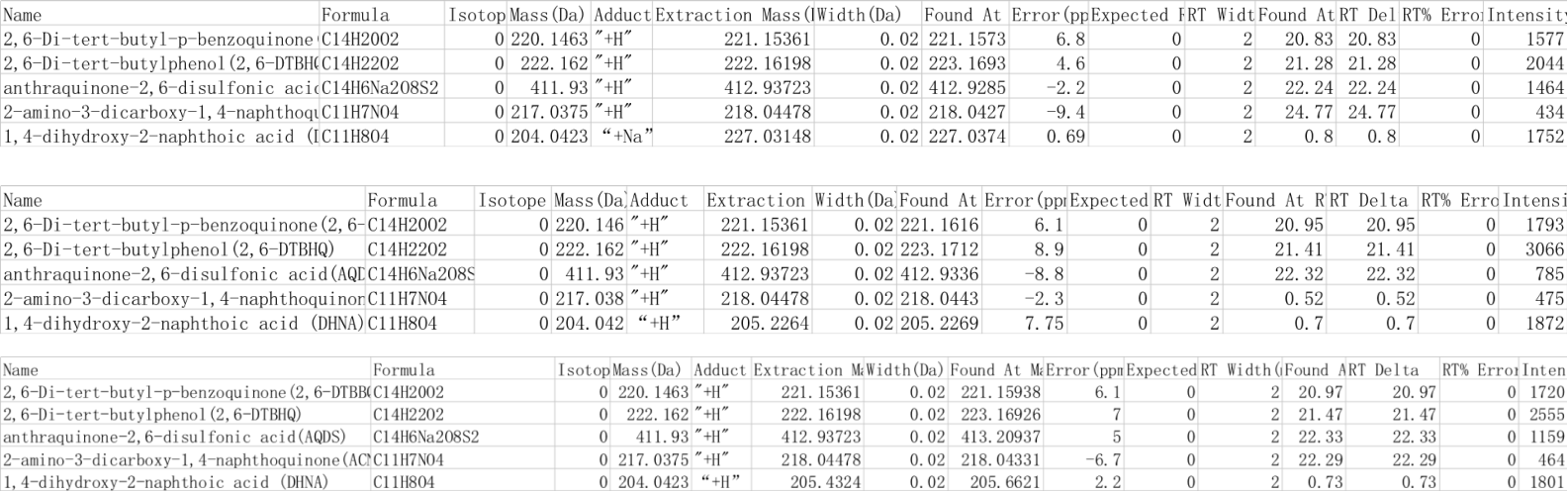


3rd


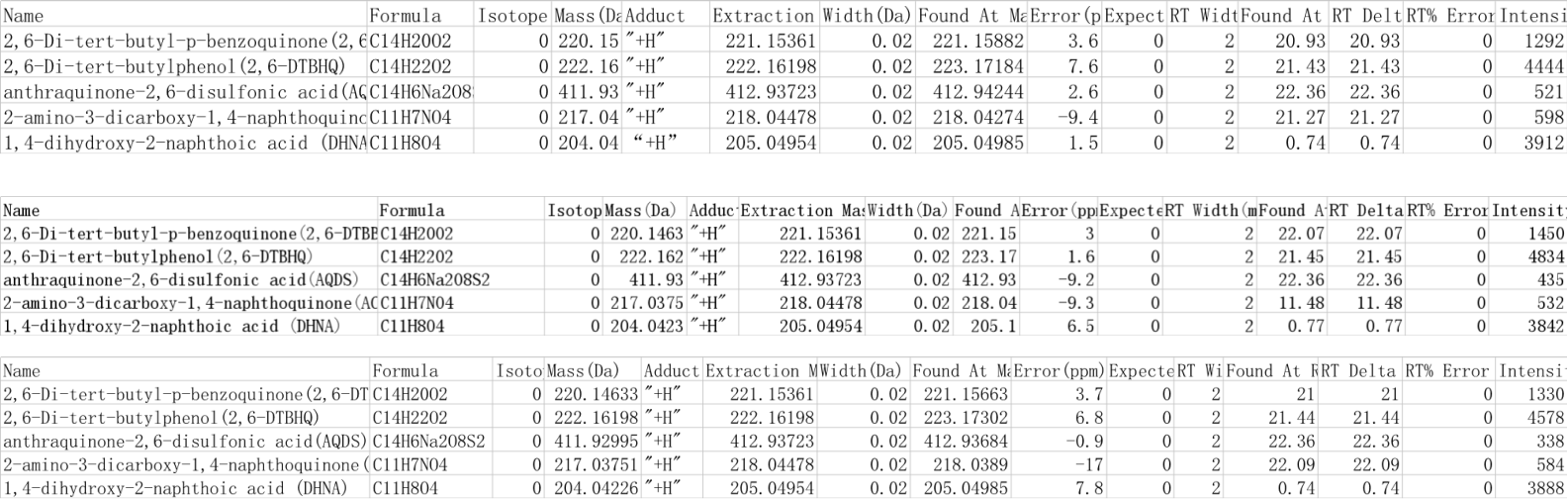


**
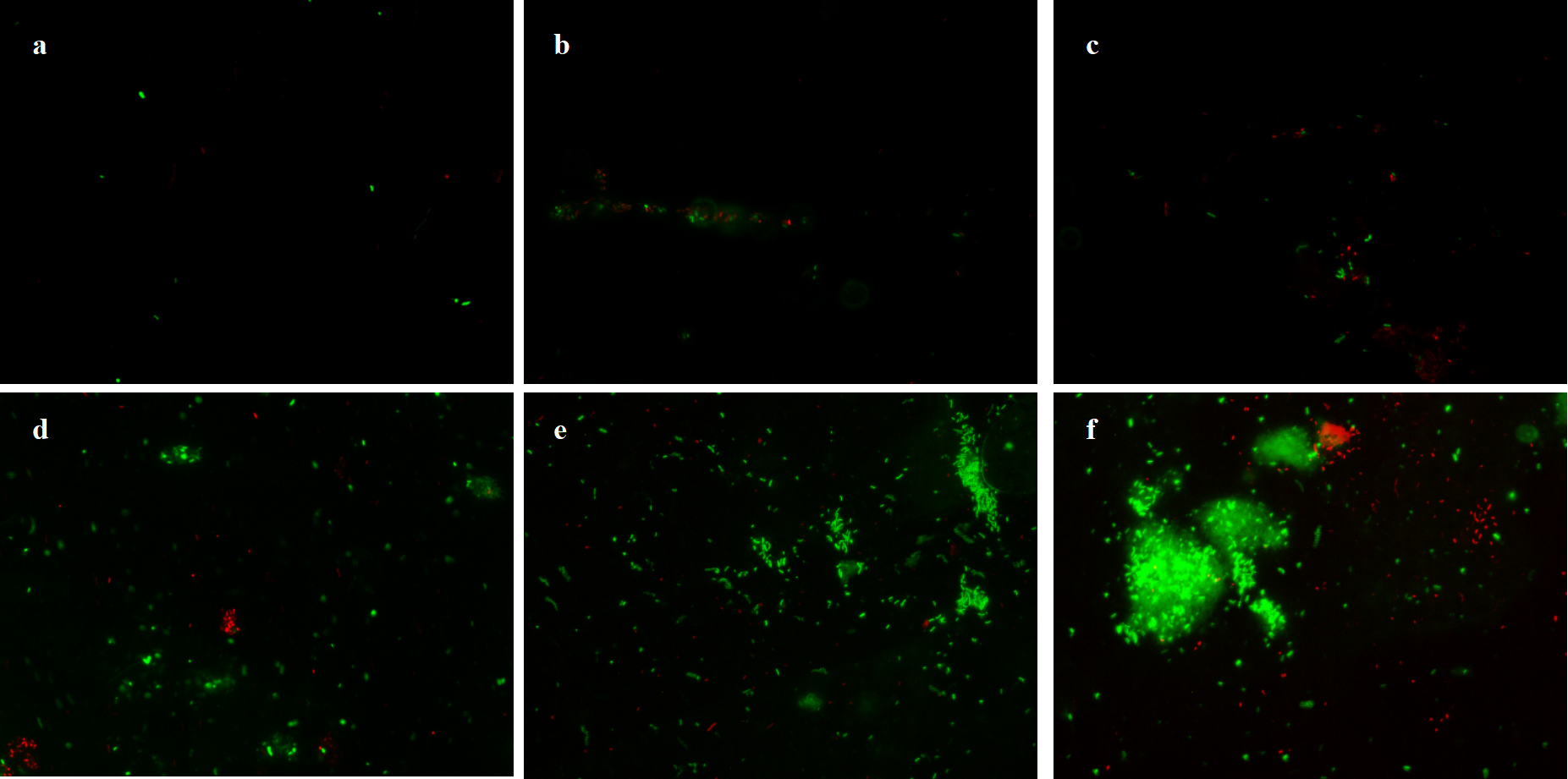
**

**Fig. S1** Images acquisition by Fluorescence Microscope, live cells were imaged as green, whereas dead cells were imaged here as red.

( **a** ) coated-MFC: 1st cycle, ( **b** ) coated-MFC: 3rd cycle, (**c**) coated-MFC: 5th cycle

( **d** ) uncoated-MFC: 1st cycle, ( **e** ) uncoated-MFC: 3rd cycle, ( **f** ) uncoated-MFC: 5th cycle.
